# Use of Frontal Lobe Hemodynamics as Reinforcement Signals to an Adaptive Controller

**DOI:** 10.1371/journal.pone.0069541

**Published:** 2013-07-22

**Authors:** Marcello M. DiStasio, Joseph T. Francis

**Affiliations:** 1 Biomedical Engineering Program, SUNY Downstate Medical Center and NYU Polytechnic, Brooklyn, New York, United States of America; 2 Department of Physiology and Pharmacology, Program in Neural and Behavioral Sciences and The Robert F. Furchgott Center for Neural and Behavioral Science at SUNY Downstate Medical Center, Brooklyn, New York, United States of America; Centre national de la recherche scientifique, France

## Abstract

Decision-making ability in the frontal lobe (among other brain structures) relies on the assignment of value to states of the animal and its environment. Then higher valued states can be pursued and lower (or negative) valued states avoided. The same principle forms the basis for computational reinforcement learning controllers, which have been fruitfully applied both as models of value estimation in the brain, and as artificial controllers in their own right. This work shows how state desirability signals decoded from frontal lobe hemodynamics, as measured with near-infrared spectroscopy (NIRS), can be applied as reinforcers to an adaptable artificial learning agent in order to guide its acquisition of skills. A set of experiments carried out on an alert macaque demonstrate that both oxy- and deoxyhemoglobin concentrations in the frontal lobe show differences in response to both primarily and secondarily desirable (versus undesirable) stimuli. This difference allows a NIRS signal classifier to serve successfully as a reinforcer for an adaptive controller performing a virtual tool-retrieval task. The agent's adaptability allows its performance to exceed the limits of the NIRS classifier decoding accuracy. We also show that decoding state desirabilities is more accurate when using relative concentrations of both oxyhemoglobin and deoxyhemoglobin, rather than either species alone.

## Introduction

Motivation for the studies in this paper stems from the search for novel approaches to brain-machine interface systems, but the reward signals investigated have broader underpinnings in the cognitive science of decision theory and value perception. Reward-modulated neural activity is an important component of conditioned behavior, motor planning, and plasticity, with ample evidence of its influence on behavior and physiology. Signals associated with reward conditions are observed in many decision making and motor planning regions of the brain, interacting with subsystems governing goal and action selection, trajectory planning, and motivation. These reward signals also offer the possibility of use as performance feedback to unsupervised computational controllers [1]. Such a controller is much more flexible in its ability to choose component actions that achieve larger goals than one trained with a supervised learning algorithm. Though this type of controller can reasonably be hypothesized to exist in the primate brain, here we demonstrate its potential use in-silico in a brain-machine interface paradigm. The objective of this work is to find a desirability signal originating in the prefrontal cortex that is recordable using near-infrared spectroscopy (NIRS), a non-invasive method for measuring blood hemoglobin concentrations. We also wanted to establish the reliability of such a signal, and prove that a reinforcement learning controller could use such realistically noisy feedback to drive useful adaptation.

### Prefrontal Cortex and Desirability Calculation

The prefrontal cortex has broad multimodal connections with many cortical association areas, along with connections to limbic cortex. It communicates with a number of important subcortical structures, including amygdala (via uncinate fasciculus), hippocampal formation (via the cingulate and parahippocampal gyri), and mediodorsal thalamus. These broad connections implicate the prefrontal cortex in motivation and complex goal-directed behavior, a hypothesis supported by lesion and functional studies [2]. The prefrontal cortex exerts its influence by way of its layer V projections to the basal ganglia (via the head of the caudate nucleus) as well as transcortically. Anatomically, the prefrontal cortex can be divided into dorsolateral (DLPFC), ventrolateral (VLPFC), dorsomedial (DMPFC) and ventromedial (VMPFC) areas. Rostral to the prefrontal cortex lies the fronto-polar cortex (FPC). The dorsal areas are most relevant for this report, since they are likely the only regions shallow enough to be probed with the light from extracranial near-infrared sources, but it important to recognize that there are significant connections between the dorsal and ventral areas. The exact functional division of these areas is only partly understood, but certain anatomical and functional connectivity patterns have been observed. DLPFC has the largest number of connections with sensory cortex, while the largest share of DMPFC connections are with motor areas [3–2].

Orchestration of cognitive branching (the process of ordering cognitive task sets for serial processing) by the fronto-polar cortex (FPC) relies in part on lateral prefrontal cortex reports of the importance of pending tasks [4]. Calculation of importance, in turn, depends on the lateral prefrontal regions' access to desirability measures for explicit stimuli or hypothesized goal outcomes. The DLPFC may thus act as an ordering memory buffer and workspace for incoming sensory information awaiting access to the cognitive stream of the prefrontal cortex. Symbolic value, or desirability, signals associated with input stimuli are likely used to establish this order. These desirability signals provide a means for prioritizing goals, predicting and avoiding poor outcomes, and generating internal drive towards specific payoffs. Such desirability signals have been reported in multiple prefrontal cortex regions, particularly in lateral and orbitofrontal cortex. In a study of different food and liquid rewards (as well as symbolic cue stimuli for them) for a monkey performing a simple delayed memory task, Watanabe [5] showed differences in the delay period activity of DLPFC neurons that correlated with the identity of the food (cabbage, potatoes, apples, raisins). In some neurons, these differences were modulated by the spatial location of the reward item (left vs. right). Thus, the prefrontal cortex may be monitoring outcomes of spatial tasks. In a promising recent study, Luu et al. have demonstrated that NIRS applied over the frontal lobe can be used to detect drink choice preferences in humans with just a single choice presentation [6].

The representation of reward has been studied more extensively in the nigrostriatal, mesolimbic, and mesocortical dopamine systems. Dopaminergic neurons in the ventral tegmental area and substantia nigra (dorsolateral portion) of monkeys exhibit phasic responses to primary rewards like food and water, as well as to auditory or visual stimuli that are learned to be predictive of reward (conditioned stimuli) [7]–[8]. Recruitment of responses to conditioned stimuli are observed after only tens of presentations, similar to the numbers needed to elicit behavioral change [9]. Midbrain dopaminergic neurons project to many areas of the brain, including the nucleus accumbens, striatum, and prefrontal cortex, suggesting that they broadcast reward prediction error (and other reward-related signals) to many disparate networks influencing cognition, motor responses, and learning. In the prefrontal cortex of primates, adenylyl cyclase activating 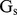-coupled D1 receptors predominate over 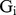-coupled D2 receptors. This suggests that *in vivo*, dopamine input provides a net activating influence in frontal areas. Interestingly, dopamine receptors tend to be found in layer V, implicating them in control of cortical output. This organization may provide a way for information related to reward to be preferentially transmitted to downstream circuits, such as those subserving motor planning. Grossly, the modulatory effect of dopamine has the effect of increasing firing, thereby contributing to increased metabolic demand and likely inducing increased local blood flow. This is in agreement with the current study's findings of increased blood flow and oxygenation fraction in response to desirable stimuli.

The DLPFC is located around the principal sulcus in monkeys and along the banks of the superior frontal sulcus in humans (Brodmann Areas 9 and 46) and it is believed to be an important mediator of polysensory working memory [10]–[11]. Synaptic dysregulation in the DLPFC is observed in schizophrenia and in mood disorders, two conditions in which value judgement is impaired. DLPFC activation has often been linked with restraint in choosing of short term rewards over delayed higher value rewards [12], particularly when favoring the delayed rewards requires instructed semantic knowledge [13]. A study of cocaine-addicted subjects, in which DLPFC experienced increased glucose metabolism when subjects were shown drug-related paraphernalia [14], provides additional support for the relationship between desirability and DLPFC activity. In a NIRS study of the prefrontal cortex of humans designed to detect emotional valence, Leon-Carrion et al. et al showed significantly increased cerebral blood oxygenation in response to a movie clip depicting sexual stimuli than to a non-sexual clip with similar complexity, both during the presentation and after the offset [15]. Observations have been made of single unit activity in DLPFC consistent with the computation of outcome desirability [16]–[17] in tasks that require these quantities to be maintained during a delay period. In human lateral prefrontal cortex, activation in fMRI is seen to increase with expected value of reward (either by increasing reward probability or magnitude) [18]. Increasing risk activates the region more if subjects were characterized as “risk seeking” rather than “risk averse” [19], indicating that hemodynamics here can be a marker for the subjective desirability of the current state of affairs as perceived by the individual.

The DLPFC receives abundant dopaminergic input from the ventral tegmentum and the substantia nigra [20]–[21] [22]. Under the hypothesis that the primary function of DLPFC is a working memory buffer input to the FPC cognitive stream, dopamine likely provides a motivating signal that is applied to processing induces increased access for reward-related stimuli. Besides the subcortical sources of dopaminergic input, the DLPFC has access to reward-related information via reciprocal connections with a number of cortical areas known to play roles in motivation and expectation of reward, including orbitofrontal cortex [23]–[24] [25] and lateral intraparietal area [26]–[27]. It also receives inputs from mediodorsal thalamus, which is thought to contribute to reinforcement [28]–[29] [30]. In 2002, Kobayashi et al. recorded spike data from DLPFC of monkeys during a spatially cued memory-guided saccade task and revealed that the firing patterns of a significant fraction of cells (>25%) contained information about reward presence [31]. During cue (200ms) and delay (900–2100ms) periods, neurons showed an increase in firing during rewarded trials versus unrewarded trials. This activity was distinct from the activity attributable to cue position, but an interesting interaction between reward presence and spatial encoding was observed: In rewarded trials neuronal information about spatial location (as measured by entropy reduction) was approximately double that in unrewarded trials, for those neurons sensitive to both reward presence and cue location. This supports the hypothesis that DLPFC activity contributes more information to spatial discrimination for more rewarding stimuli.

**Figure 1:**
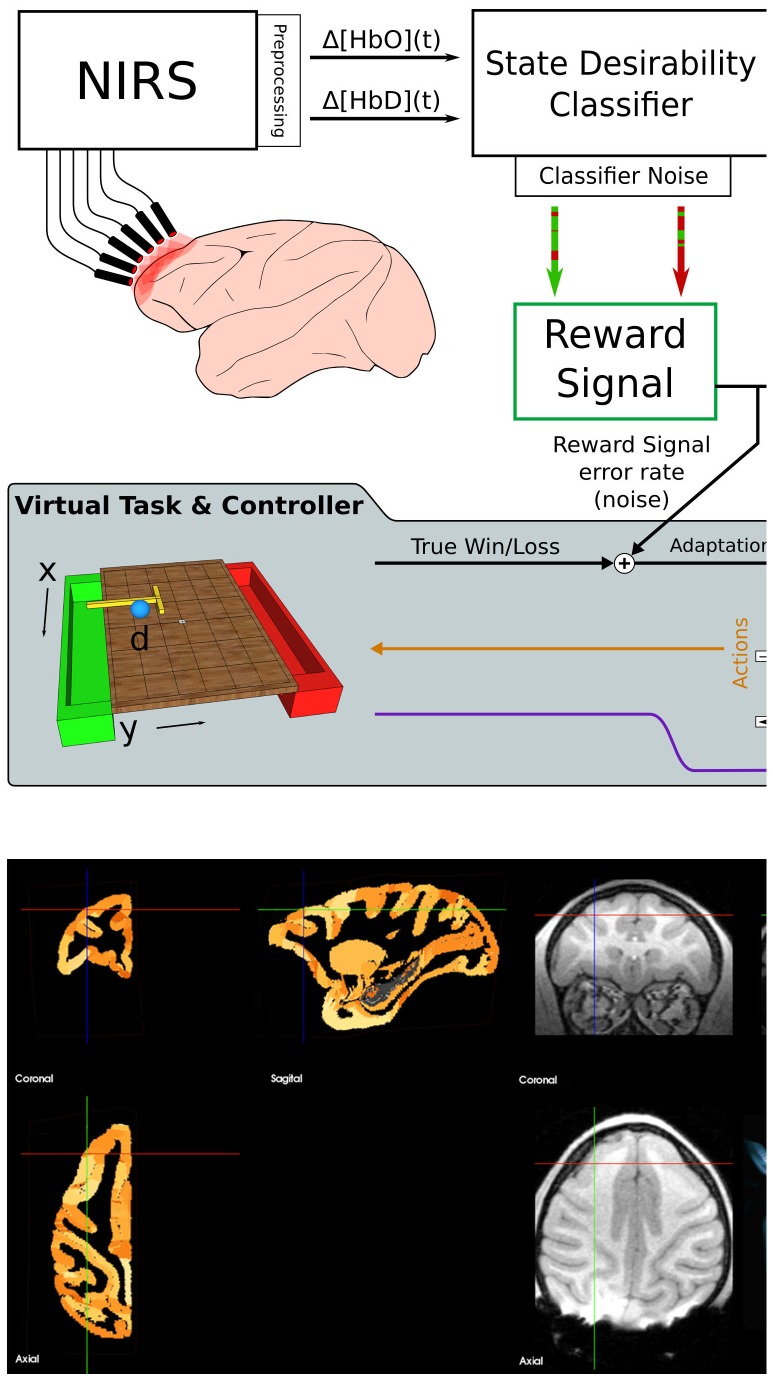
Experiment and Model Summary. **A:** The reward signal is derived from the subject's frontal lobe hemodynamics. The Δ[HbO] and Δ[HbD] signals recorded at times around events are classified using a support vector machine (SVM) in order to read out their prediction about the subjective desirability of the event. Any classifier is subject to some misclassification noise (green arrow with red imperfections, and vice versa) so the RL agent that uses this signal as reward information must be robust to occasional misclassifications. Gray inset: The error rates achieved by the SVM classifier in this study were added to the win/loss feedback to a model task in which the reinforcement learning agent had to select actions to be taken by a rake tool in order to achieve the goal of pulling a pellet off of the front side of a table, without knocking it off the back side. The adaptation of the action values for the most recently observed state (and thus the adaptation of the agent's control policy in subsequent visits to that state) is dictated by the reward signal. The agent learns to select the action with the highest expected return, given the current state (i.e. the locations of the pellet and rake tool). **B:** Brain MRI of the rhesus macaque used in this study. The T1-weighted MRI image (right panel) was registered to a standard atlas (left panel) to locate the DLPFC region of cortex (indicated by the crosshairs). Skull landmarks were then used to localize and place probe guides during implantation. The lower right subpanel shows a 3D reconstruction of the subject's head with dots at the locations of the NIRS probes used as sources (purple) and detectors (red).

In studying a task in which the relationships between visual cue stimuli, motor responses, and reward conditions were varied, Matsumoto et al. demonstrated that neurons in the monkey medial and lateral prefrontal cortex have firing activity that can be related to any combination of (cue, response, reward), with pure responses to reward condition most prevalent (25% of recorded cells) [32]. The recordings were made around the principle sulcus, close to the area of interest in the present study as per Figure 1B.

Activity related to purely perceptual decision making has also been observed in the dorsolateral prefrontal cortex using fMRI and single unit recordings [33]. Neurons in DLPFC have been observed to maintain spiking during the delay period between instruction and execution of a movement, in a stimulus- or location-selective manner [34]–[35] [36]–[37]. The discriminations studied in these experiments are not based on reward value, but simply on the ability to differentiate between noisy stimuli. This activity too was soon found to be modulated by the opportunity for reward. Their firing rate during a memory period between cues and saccades to targets is higher during trials with a large reward than during trials with a small reward [38]. Notably, this differential firing did not occur at reward cue presentation, but during the memory period when both reward and spatial cue stimuli were absent. Though the contingencies for DLPFC activation are complex, it appears likely that this region is engaged in processesing reward value or desirability of stimulus representations in working memory.

### Reinforcement Learning Overview

Reinforcement learning (RL) algorithms make use of three elements: *states*, or information about the environment from sensors, *actions*, or commands passed to actuators that interact with the environment, and *reinforcement signals*, which drive adaptation of the algorithm in its selection of appropriate actions. An RL controller learns a policy of action selections as it practices interacting with its environment. Traditional BMIs focus on decoding neural signals without learning state-contingent policies. They are therefore relatively inflexible when compared with BMIs that make use of artificial intelligence or machine learning to select actions that best accomplish the users' objectives. We believe prosthetic and other human-robot interactions have the potential for much more intuitive use if adaptive algorithms are used as controllers.

The task of an intelligent BMI controller can be separated into two domains: 1) *decoding* the user's instructions from the neural interface and 2) *policy learning*, or the selection of the appropriate computer/robot commands for the present task and situation. In an RL controller, the computer agent would decode the user's satisfaction with its performance and attempt to learn a policy that is most pleasing to the user, continually updating its behavior as the environment and needs of the user change. The user and agent would thus form a coadaptive system as each learned to work with the other towards the shared goal of maximizing the user's satisfaction. The possibility of using prefrontal cortex signals as reinforcement signals to drive policy learning is an attractive one, but is tempered by biological and sensor noise, along with difficulty in interpretation of prefrontal cortex activity. It is of central importance to select control algorithms that are robust to these sources of uncertainty in a potential reward signal. Furthermore, the environmental information gathered by sensors and available to the controller represents only a partial representation of the real external state. Finally, actions selected may not always have the intended effect, since actuators are unreliable and are subject to unpredictable contingencies in the environment. Control algorithms handle these error sources with varying tradeoffs between adaptability, training speed, and complexity. For our tests of a controller's performance when given feedback with noise equal to our observed NIRS DLPFC decoding inaccuracy, we chose the 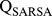 learning algorithm.


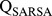
, an implementation of the temporal difference (TD 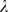) family of RL algorithms, was selected for the present work due its good performance when faced with partially observable but non-deterministic outcomes. Like other temporal difference methods, 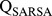 is a method of reward prediction for learning a policy to be applied in a Markov decision process (MDP) [39]. Modeling BMI control as an MDP seems appropriate, since interfaces would be expected to be continually adapting over years of use, and present states could be taken as independent of states encountered in the distant past. 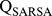 is an on-policy learner, meaning that it learns the value of actions that are actually chosen, as compared to off-policy learning, which makes value calculations based on hypothetical choices, and then selects the best ones to execute. In general, the current on-policy RL agents are more data efficient than off-policy agents [40].

Watkins and Dayan (1992) proved that in an MDP with a finite set of states and actions, learning agents of this class converge to the optimal policy, assuming that all actions are repeatedly sampled from all states. This proof is only valid for single step updating of action values (as opposed to the multi-step history updates employed by the algorithm explored in this work, which learns more quickly). Nonetheless, multi-step 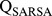
's convergence to policies with excellent performance has been empirically observed in many applications [40]–[41] [42]–[43]. Furthermore, 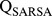 is often endowed with a little bit of “jitter” in its action selection policy, in order to explore the possibility of policy improvement, at the cost of stability and speed of convergence. By managing the history length and jitter parameters of a 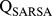 agent, satisfactory levels of learning speed and performance can often be achieved. In fact, 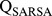 is often found to learn quite rapidly, when compared with other artificial intelligence methods [44]. 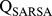 controllers are well-suited to applications in which both sensor and actuator noise are present, there are hidden states of the environment not observable by the controller, and the sensor/action cycles are asynchronous [41]. These are conditions likely to be in place in human prosthetic control applications. Developing shared control of prosthetics between the users' brains and the computer controllers also requires direct feedback from the user to the controller about satisfaction with device performance. This feedback is also subject to noise, which motivates the current study.

### Study Objective: Using Desirability Signals in BMI Systems

A BMI operating under the control of a reinforcement learning (RL) agent requires defined rewards whose maximization is the agent's goal. In this work, we aimed to establish the feasibility of decoding a desirability signal associated with conditioned stimuli from the rhesus macaque DLPFC that could function as a reinforcement signal input to a 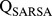 controller. To acquire this signal, we used NIRS, a non-invasive technique that probes regional cerebral blood flow by measuring the reflectance, and calculating absorbance, of infrared light. The absorbance waveforms were classified according to their association with high and low desirability outcomes. The error rate in this classification was then applied to the reward signal in a model 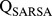 learning application. In so doing, we aim to demonstrate that such an RL controller can drive useful learning when provided with a realistically noisy cerebrally-derived reinforcement signal. The general approach used in this study for validating the signal, and model used as a testbed for its use in an RL controller are illustrated in Figure 1A. This work is part of a continuing investigation into the use of reinforcement learning agents as controllers in brain-machine interfaces, using multiple brain signal sources [45]–[46] [47].

## Materials and Methods

### Ethics Statement

This study was performed in strict accordance with the recommendations in the Guide for the Care and Use of Laboratory Animals of the National Institutes of Health. The protocol was approved by the Institutional Animal Care and Use Committee of SUNY Downstate Medical Center (Imaging Protocol Number: 11–102–42; Experimental Protocol Number: 06–465–10). Implantation surgery and MRI imaging were performed under ketamine/isoflurane anesthesia, and every effort was made to minimize suffering.

Overall care was managed by the DLAR (Division of Laboratory Animal Resources) at SUNY Downstate Medical Center. The subject was housed in a large individual enclosure with other animals visible in the room, and looked after daily by the senior DLAR staff, who also weighed the subject weekly and updated daily feedings in order to maintain weight. The in-house veterinary doctor checked the subject before the start of the study, and performed blood tests and physical examinations as needed. The subject was given weekly fruit or dry treats as a means of enrichment and novelty. In collaboration with DLAR, we have attempted to offer as humane treatment of our subject as possible, and we believe that the standard of animal care and welfare in our lab exceeds national guidelines.

### Surgery and Instrumentation

A 3 year old male rhesus macaque monkey weighing : 5.3 kg was used in this study. A series of T1-weighted MRI images (coronal slices) of the head of the anesthetized animal were acquired on a 3T Siemens scanner while it was mounted in a stereotaxic frame in the sphinx position (which improves magnetic field homogeneity throughout the brain volume [48]). Vitamin E fiducial markers were affixed to the frame, and to the animal's head at nasion, inion, and at the mastoid processes. The image with the best contrast homogeneity was selected and used to calculate distances between the dorsolateral prefrontal cortex (DLPFC) and various skull locations relative to the fiducial markers. The image was registered onto a standard rhesus brain (the MNI rhesus atlas, composite of 7 adult rhesus macaques [49]) via affine transformation (BioImage Suite software [50]–[51]). In this standard space it could be visualized and navigated through in relation to a standard atlas, which helped localize the anterior extreme of the principal sulcus. The markers were replaced on the stereotaxic frame during the surgery and guided the final choice for fixation location of the PVC guides for the NIRS optodes (see Figure 1B).

During this surgery the frontal portion of the skull was exposed, cleaned, and dried. A series of fixation screws were implanted in the bone, and a thin layer of translucent acrylic was applied in an adaptation of a technique heretofore only attempted in rats [52]. The PVC NIRS guides were placed over the cortical region of interest and allowed to adhere to the acrylic until it hardened. Then pallicose bone cement was used to surround the guides and secure them to the screws. During the procedure, two intracortical microelectrode arrays were implanted in the cortex (in the hand regions of both primary motor cortex and primary somatosensory cortex, following a previously established procedure [53]), and a depth electrode array was placed in the ventral posterior lateral nucleus of the thalamus. The connectors for these, along with the NIRS guides were integrated into a single external recording apparatus by applying a top layer of opaque acrylic dental cement to seal the implant to the surrounding hardware and skin margins.

On each day of recording, the PVC guides were cleaned and the optical fiber probes (2 sources and 4 detectors) from the NIRS instrument were placed into their assigned guides. The distances between source probes and their associated detectors were approximately 1cm. These distances correspond very roughly to 300mm–1cm tissue penetration depth, according to the 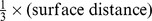 rule as measured by Cui et al. [54].

The NIRS acquisition was done with a NIRScout system from NIRx Medical Technologies (Glen Head, NY). This system is capable of capturing data from 16 sources and 24 detectors, but only a subset (2 sources and 4 detectors that fit the implanted guides) were used in the present work. “Sham” test recordings with no animal were made in the chamber with the video screen updating to make sure that light from the screen or other ambient sources did not affect the measurements. No changes in the recordings were observed on screen updates or with changes in the chamber lighting.

Stationarity of the head was maintained with a fixed head post attached to the parietal bone. The monkey was seated in a chair facing a video screen on which the visual cues were presented. NIRS data for wavelengths 760nm and 850nm was collected for each source-detector pair at a frame rate of 6Hz. Time-synchronized video was captured throughout a subset of the experiments.

### Experimental Protocol

#### Conditioning stimuli

**Figure 2:**
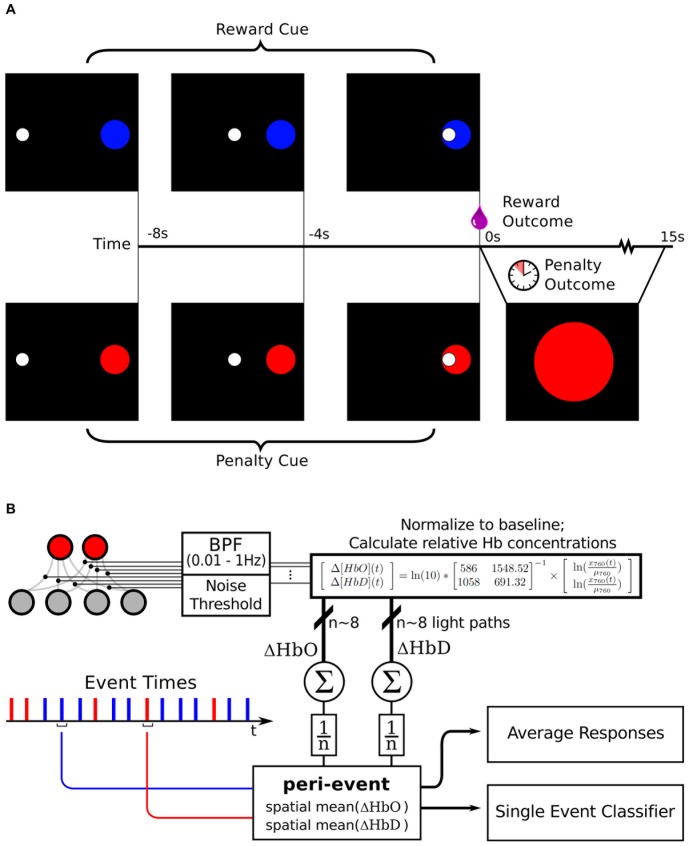
Experimental protocol and NIRS data processing summary. (A) Visual stimuli: The cue target and cursor appear 8s before the predetermined outcome (which is chosen randomly on each trial). Target locations vary around a circle of fixed radius 10cm. The cursor moves with a fixed speed towards the target, and when it reaches the target, the outcome stimulus is delivered. A reward outcome is 0.5mL pomegranate juice delivered through the sipper tube. A penalty outcome is a 15s period of waiting in which a colored disc matching the penalty cue was presented in the center of the screen. A random interval (mean 20s) was then enforced before the start of the next trial. In a subset of experiments, the color significance was reversed. (B) Data is analyzed from all pair-wise combinations of sources (red dots) and detectors (grey dots). Each source-detector pair time series of 760 and 850nm readings that exceeds a signal/noise threshold is band-pass filtered and used to compute ΔHbO and ΔHbD time series for that light path. These time series are then averaged across light paths. The path-means in the period around the reward stimulus and penalty stimulus events are then analyzed further, either as peri-event means, or by classification of single event peri-event path mean waveforms.

The monkey was placed on controlled water access for 16–24 hours before each day of experiments. For each trial, after a 10s baseline (blank screen), the monkey was presented with a visual display of a single white disc “cursor” in the center of the screen and a colored disc “target” 10cm away (see Figure 2A). These serve as a cue for the animal, indicating the nature and latency of an upcoming outcome stimulus. The cursor moves in 16 steps towards the target (0.5s per step; 8s total trial duration). The outcome of the trial was dictated by the color of the target (blue 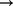 reward; red 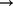 penalty). A custom-designed program written in Python was used to control the visual cues and the delivery of liquid rewards, as well as to generate serial data event signals that were logged by the NIRS acquisition system. The animal was exposed to cue-outcome pairings for ten 45 minutes sessions (: 30 rewards and : 30 penalties each) in order to allow the animal to establish the association between cues and outcome.

Next, a series of NIRS recordings was done while the animal was repeatedly presented with these same pairings, with desirable and undesirable outcomes intermixed. In 75% of the experimental sessions, the blue target indicated that when the cursor reached the target, a pomegranate juice reward (0.25mL) would be delivered and the red target indicated that when the cursor reached the target, a time-out period would be enforced. The time-out was a 15s duration in which the cursor and target disappeared and a fixed red disc appeared in the middle of the screen before the start of the next trial's 10s baseline period. In the remaining 25% of the experimental sessions the significance of the red and blue targets was reversed (red = reward; blue = time-out). The outcomes have intrinsic desirability (appetitive value of juice and delay in obtaining more liquid for a thirsty animal). The cues come to have secondary desirability through their repeated pairing with the outcomes. The monkey was over-trained on both these stimulus sequences (10 sessions of 30–50 presentations each), and then NIRS recordings were made during 20 experimental sessions of 40 min duration, comprising approximately 60 trials each. Thus the cursor and target form a conditioned stimulus, and the juice reward or time-out penalty form an unconditioned stimulus.

#### Unexpected stimuli

In an earlier set of experiments (n = 20), two different liquids (pomegranate juice and vinegar) were delivered to the animal while it was seated in the chair viewing a fixation cross. Without any predictive stimuli, 1mL of either juice or vinegar was delivered through the sipper tube. The tube was placed onto the tongue such that both liquids elicited similar swallowing movements. Approximately 20 deliveries were made during each 1 hour experiment. NIRS data was recorded throughout these experiments and event times logged.

The animal's preference for pomegranate juice was established previously by simultaneously providing 200mL of both liquids for free consumption in the home cage for five 20 minute sessions, during which it consumed an average of 105mL of juice and 0mL of vinegar (see Results).

### Data Analysis

#### Preprocessing and relative hemoglobin calculations

The hemodynamic signals evaluated in this work are the concentration differences (relative to a baseline period) of oxyhemoglobin (Δ[HbO]), deoxyhemoglobin (Δ [HbD]), and total hemoglobin (Δ [HbTot]). The baseline periods are the intervals of quiet resting (with no reward-relevant stimuli) immediately before the onset of the first stimulus in the trial.

Each NIRS source-detector pair forms a channel, corresponding to a distinct light path through the tissue. All channel data were band-pass filtered in the range 0.01–1 Hz, in order to remove artifacts due to drift, heart rate, and breathing. Any channel with a signal-to-noise ratio 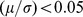 for either wavelength was considered to be too noisy and discarded.

For each trial, the NIRS data from the 10s prior to the cue presentation was used as “baseline”, and the reported hemoglobin concentrations are relative to this baseline for each trial. This is done in order to further normalize for long-term trends in hemodynamics, and extract components of the signal that are truly event-related. NIRS detector data acquired during the trial (i.e. between cue onset and 15s after outcome offset) was then used to calculate oxyhemoglobin and deoxyhemoglobin concentrations relative to the baseline period. The relative concentrations are computed from the detector data for the two wavelengths according to:
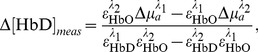
(1a)

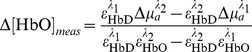
(1b)Where 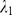 and 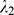 are the wavelengths of light used (760nm and 850nm respectively), 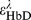 and 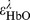 are the extinction coefficients for the two chromophores of interest (HbO and HbD) at wavelength 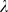, and 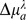 is the observed change in absorption coefficient at wavelength 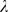 [55]. We use the recorded absorbances at each time point 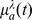 normalized to their baseline means 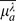 as the change in absorption for that time point 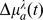. Then, reformatting equations 1a and 1b into a matrix equation, and incorporating the known extinction coefficients for 760nm and 850nm light (
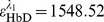
, 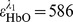, 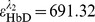, 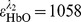; see http://omlc.ogi.edu/spectra/hemoglobin by S. Prahl, also [56]) yields
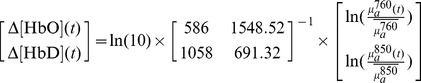
(2)which was the actual calculation made during preprocessing (see Figure 2). This yields Δ [HbO(t)] and Δ [HbD(t)], the concentration changes relative to baseline in oxy- and deoxyhemoglobin, respectively, for each channel, assuming a path length of 1cm each.

Δ [HbO(t)] and Δ [HbD(t)] were then averaged across channels for each time step from cue onset to 15s after outcome offset. These average Δ [HbO(t)] and Δ [HbD(t)] time series for each event were then analyzed in two ways: mean responses to multiple presentations of reward and penalty events, and single trial classification of events as either rewarded or penalized. Mean responses and standard error of the mean to rewarded vs. penalized events were computed, and significance levels at each time step were determined with Welch's t-test.

#### Peri-stimulus statistics and analysis

In order to characterize the first order statistics of the NIRS signals around desirable and undesirable stimulus times, the trials were separated according to their known outcome, and means and standard errors of the means (SEM) were computed for each peri-event time step. This analysis was carried out for cued trials and uncued trials. In order to test for the effects of motion artifacts, video recordings of the animal€s face were taken during a subset of cued experiments (n = 4). The video was time-synchronized to the NIRS recording. This video was analyzed manually in order to tag trials in which the animal exhibited overt facial movements. Head movement was prevented by the fixed head post restraint. All frames of video around trial times were reviewed, and if the tongue or teeth were visible or lip movement of : 1.5cm was observed at any time during trial, the trial was tagged as a “movement” trial. These trials were then set aside and averaged separately from the “non-movement” trials.

#### Single trial classification

Single trial NIRS data were classified using a support vector machine (SVM) classifier. SVMs are a generalization of the technique of linear decision boundary search to situations in which the two classes of interest are not linearly separable. By transforming the feature space, SVMs are able to find discriminating hyperplanes that can separate examples from classes that are in overlapping regions of the original space. This proves to be the case for the peri-event Δ [HbO] and Δ [HbD] signals recorded in this study, motivating the use of SVMs for classification. SVMs attempt to find the maximum-margin hyperplane that separates examples of the two classes in transformed feature space. Stated concretely, SVMs search for the hyperplane 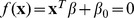 under the constraint.
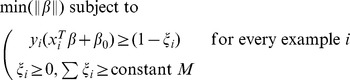
(3)where 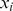 is an example data vector and 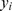 is its associated class label in {−1,1}. 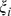 is a slack variable associated with each training example that dictates how “fuzzy” the classifier margin is allowed to be. The total proportional amount by which examples may be on the wrong side of their margin is bounded by the constant 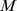. This minimization can be formulated as a convex optimization problem, allowing the global optimum 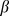 and 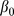 to be obtained. These define the hyperplane that creates the largest margin between training examples of the two classes. The margin is the distance from the hyperplane to the nearest example. Thus, not all examples contribute to the definition of the optimal hyperplane, allowing the SVM to be computed efficiently. SVMs are relatively good at dealing with high dimensional data classification problems as well [57].

The performance of the classifiers were evaluated with a jack-knife cross validation scheme: For each of 100 rounds, a randomly selected trial is set aside as a test example, the SVM is trained on the remainder of the data, and the trained SVM is used to classify the test example as a reward trial or penalty trial. The average classification performance on all test examples is taken as a measure of the SVMs ability to generalize to new trial data to which it is naïve. It was also a goal of this study to determine which hemodynamic signals would provide the most information about stimulus desirabilities, so separate SVM classifiers were trained and tested using only Δ [HbO], only Δ [HbD], or Δ [HbO] and Δ [HbD] together to form 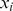.

### Reinforcement Learning Model Task

The model task consists of a 7×7 grid in a plane with a pellet located at one of the grid vertices, and a rake tool that is represented by a T shape in the plane. The rake can only move the pellet forward and backward, not side-to-side, and does so only if the pellet location intersects with one of the arms of the head (which together span 3 grid squares). The state of the model environment is represented completely by the pellet location on the table, and the 1-D direction (in front/behind) and distance from the pellet to the rake: 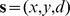. The actions available to the agent were movements in the plane in each of four directions: 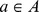, 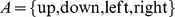.

The pellet location is initialized to the center of the grid on each trial. If the rake pushes the pellet off the back of the grid, the reward value for the trial is −0.2, and if the rake pulls the pellet of the front of the grid, the reward value is 1. Achieving a reward requires moving the rake to the side before moving it back, so that it doesn't push the pellet backwards, followed by a movement back to the center and a pull to the front of the grid.

It should be noted here that finding the optimal control strategy for this task requires the agent to evaluate sequences of actions based on delayed rewards. Because the task-specific rewards are only delivered at the end points of executed trajectories, when the pellet falls off the table, the agent must maintain a memory trace of it’s action selections. Since the finite-numbered states and rewards in this task depend only on their immediate antecedents, they can be said to form a finite Markov decision process.

#### Q_SARSA_ algorithm


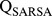
 is an “on policy” method of value estimation, meaning that the agent's paths of exploration of the value landscape are bound by the actions it actually chooses to implement (the policy). This is not necessarily restrictive, and under any policy that allows for every path to be visited infinitely often (given infinite time), the value function estimate can be shown to converge to the true value function. In our implementation, this requirement is satisfied by a policy of 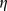-greedy action selection, in which the highest value action 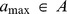, where A is the set of all possible actions, is selected with probability 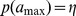 (usually large), and all other actions 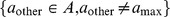 are selected with uniform probability.
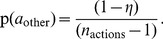
(4)


We'll call this policy 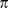. 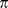 is the complete description of the actions to be chosen for all states based on their estimated values (which we will store in matrix 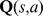) and the 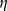-greedy action selection rule. Thus 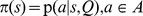. 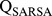 attempts to learn the best estimate of (state, action) values 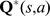 by updating its running estimate 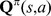 as the agent follows policy 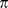. It accomplishes this by use of the temporal difference rule TD(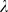), which iteratively updates 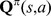 with weighted contributions from newly received rewards and prior value estimates. This update procedure thus takes the form of the algorithm shown in Figure 7.11, Section 7.5 of [58].

In order to deal with stochastic reward signals of the type delivered by the NIRS classifier, the 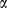 parameter (learning rate; see [58]) is annealed (decreased) according to the number of times each particular 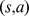 pair has been visited, so that realized rewards contribute less and less to the running estimate, thus attenuating unstable fluctuations based on an inconsistent reward signal (see Discussion).

The model rake task has 1183 possible states, and 4 possible actions. The 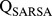 algorithm was run on this model task for 200,000 time steps, starting a new trial with each terminal state (front edge or back edge) and using 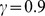 and 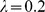.

An experiment of this type was run on each of the reward classification accuracies {0.55, 0.60, 0.65, 0.7, 0.75, 0.8, 0.85, 0.9, 0.95, 1.0}. For each experiment, a record of rewards (including negative rewards, or penalties) was kept. The running average of the fraction of trial outcomes that were true positive rewards (and notnegative penalties) was calculated as a summary of the agent's performance. The simulation and 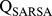 algorithm were implemented in MATLAB (Mathworks Inc., Natick, MA).

## Results

### Decoding Preferences from NIRS recordings

#### Unexpected liquid rewards and liquid penalties

**Figure 3:**
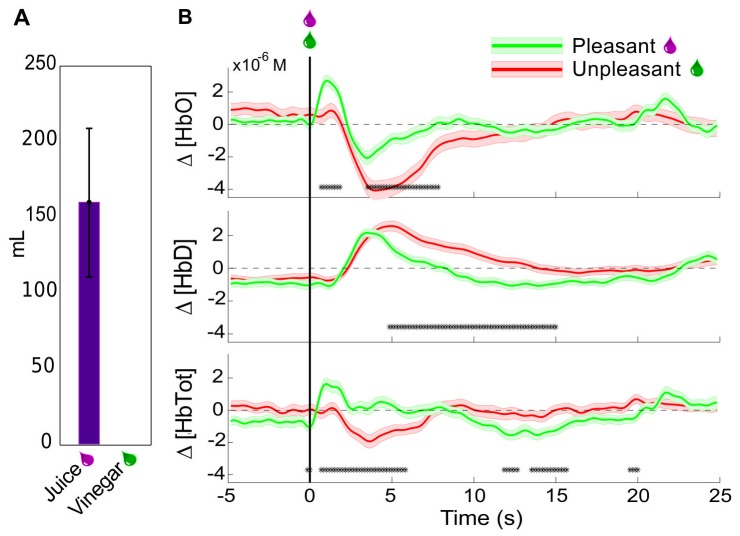
Hemodynamic responses to uncued rewards and penalties. (A) Mean±SEM amount of liquids consumed when both were presented ad libitum simultaneously for 20 minutes in the animal's home enclosure on 3 days. No vinegar was consumed on any day. (B) Mean±SEM Peri-event changes in Δ[HbO], Δ[HbD], and Δ[HbTot] relative to baseline for unexpected delivery of 0.5mL of pleasant liquids (pomegranate juice or water) or unpleasant liquid (vinegar). Events delivered at pseudo-random intervals (min 40s). Asterisks indicate times at which the responses in pleasant and unpleasant trials were significantly different (Welchs t-test, p<0.05). (n = 121 rewards; n = 88 penalties).

When given free access to juice and vinegar, the monkey immediately began drinking the juice at each presentation, and consumed an average of 160mL of juice over the interval. In contrast, after testing the spout on the reservoir containing vinegar, the monkey withdrew quickly, and never consumed any of the liquid. When vinegar was directly applied in the mouth with a dropper, the monkey attempted to prevent the application, and vocalized more frequently than normally observed. The prefrontal hemodynamic responses to unexpected delivery of pleasurable and aversive liquids were then tested by head-posting the animal in an experiment chair and positioning it with a sipper tube in its mouth. Then juice or vinegar were delivered in 0.5mL boluses onto the tongue without any other predictive stimuli at pseudo-random (Poisson distributed with mean interval 60s, but with minimum interval 40s) times. These were delivered in blocks of ∼60 trials, with a single type of liquid in each block, in order to minimize the possible mixing of taste stimuli. Δ [HbTot] and Δ [HbO], but not Δ [HbD] were observed to rise significantly more in the period immediately following juice delivery versus vinegar (Figure 3). A : 5s decrease in oxyhemoglobin relative to pre-event baseline was observed for both pleasant and unpleasant stimuli, but was significantly more pronounced for the unpleasant stimulus. Thus, in this biphasic oxyhemoglobin response, both phases showed modulation by the desirability of the liquid stimulus. The deoxyhemoglobin concentration changes around the events were the same for both types of stimuli for the first 5s after presentation, but the second phase, a slow return to baseline, was prolonged for the undesirable stimuli relative to the desirable ones. The total hemoglobin changes naturally show a combination of these patterns, with an initial rise in mean following only desirable stimuli. The decrease in total hemoglobin from : 2s–6s brings the value to baseline for desirable stimuli, and down to a deficit relative to baseline for undesirable stimuli.

The approximately 15s event-related perturbation and return to baseline corresponds to that observed in prior NIRS studies of cortical activation in response to motor imagery [59]–[60], motor tasks [61]–[62] [63], and working memory activation [64] in humans. The more rapid switch between Δ [HbO] increase and decrease than is observed in other studies is likely due to the brief nature of the unconditioned stimuli used in this study.

#### Cued rewards and penalties

**Figure 4:**
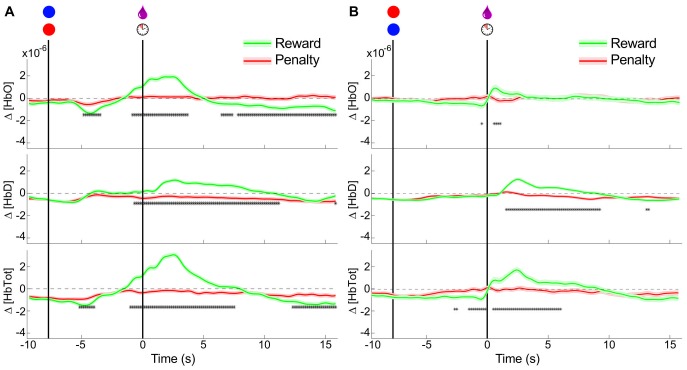
Hemodynamic responses to cued rewards and penalties. Mean±SEM Peri-event changes in Δ[HbO], Δ[HbD], and Δ[HbTot] relative to baseline for cued delivery of 0.5mL of reward liquid (pomegranate juice) or enforcement of a penalty time-out period (10s of presentation of a stationary red disc). Asterisks indicate times at which the rewarded and penalized trials were significantly different (Welch's t-test, p<0.05). (A) NIRS signals around cue and outcome presentation for blue cues predicting rewards and red cues predicting penalties (n = 658 rewards; n = 588 penalties). (B) NIRS signals around cue and outcome presentation with the color significance reversed: blue cues predict penalties and red cues predict rewards (n = 118 rewards; n = 95 penalties).

The study also attempted to determine whether the separability in hemodynamic responses to rewarding and aversive stimuli could be translated to conditioned stimulus types, or whether it depended on the intrinsic appetitive value of the stimuli. The observed post-event hemodynamic changes agree with those observed in the un-cued trials (i.e. Δ [HbO] is increased following desirable stimulus delivery, but not undesirable stimulus delivery). A significant anticipatory rise in both Δ [HbO] and Δ [HbD] immediately prior to desirable stimulus delivery is also observed, further differentiating rewarded and penalized trials. A decrease in Δ [HbO] relative to pre-trial baseline was seen for approximately 3 to 5 seconds following the cue presentation for both rewarded and penalized trials, indicating the animals awareness of both types of cue. This decrease was more pronounced for rewarded trials. There is also a slight decrease in Δ [HbD] around the cue presentation, nearly identical for both types of cue. These results are summarized in the left panel of Figure 4.

Taken together, the cued and uncued trial results indicate an increase in total blood flow in the prefrontal cortex in response to primarily desirable stimuli, comprising an increase in both Δ [HbO] and Δ [HbD]. A decrease in total blood flow is observed in response to secondarily rewarding stimuli, mostly due to the decrease in Δ [HbO]. Changes in response to undesirable stimuli are much less pronounced, but include a small decrease in Δ [HbO] following cue presentation and at the time of outcome presentation.

A post-outcome rise in Δ [HbTot] contributed by both Δ [HbO] and Δ [HbD] indicates an increase in regional cerebral blood volume at this time, which would be expected to accompany increased neural activity during this period under standard models of neurovascular coupling [65]. The increase in measured Δ [HbD] during this period is equivocal regarding cerebral metabolic rate of oxygen, which is expected to more closely parallel neural activation [66]. Nonetheless, the regional cerebral blood volume increase in response to desirable outcomes likely corresponds to the known positive modulation of prefrontal neural firing in response to rewarding stimuli [38]. A smaller negative perturbation in Δ [HbO] and Δ [HbTot] is observed during the period between cue and outcome.

#### Color-reversed trials

In order to control for the possibility that the differential activity observed around the visual cue stimulus was based only on the color, experiments were run in which the reward-predictive significance of the target colors was switched (Red = Reward, Blue = Penalty). After retraining the animal on these reversed cues for three days, NIRS recordings were made. The same qualitative pattern was observed as in the original color cue scheme: an anticipatory decrease in Δ [HbO] for both trial types followed by an outcome-selective increase in both Δ [HbO] and Δ [HbD] (see right panel of Figure 4). The amplitudes of the responses in the color-reversed experiments were smaller than for the original color scheme, and there was less significant differentiation between the trial types based on the cue alone. This may be attributed to the residual effect of the original color scheme creating some decreased certainty in the cue significance. It may also be due to a long-term attenuation of the response with repeated exposure, since the reversal experiments were done after the first color scheme had been established. Nonetheless, outcome discriminability does appear to be independent of the color of visual stimuli.

#### Comparison of separated motion artifact trials

**Figure 5:**
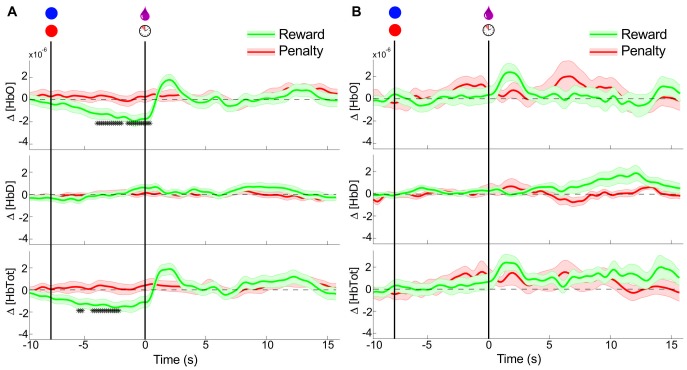
Comparison between trials with and without significant facial movements. Conventions as in Figure 4. (A) Peri-event NIRS signals for trials in which no movement was identified on video. (n = 62 rewards; n = 69 penalties) (B) Peri-event NIRS signals for trials in which overt facial movements were observed; see Materials and Methods (n = 35 rewards; n = 24 penalties).

Though little head motion was possible due to the head-restraining post, a possible source of task-related artifact in the NIRS signals is the movement of the facial and scalp muscles. No motion of the NIRS probes was observed during lip and tongue movements, but in order to rule out the possibility of the observed signal changes being caused by these, video was captured during a subset of the experiments. Trials in which overt facial or tongue movements were observed (defined as visibility of the teeth or tongue at any point during the trial, or movement of the lips >2cm) were separated. These trials, and those in which no movement was observed were analyzed separately, and the results shown in Figure 5.

The similarity of the presumed hemodynamic changes in the trials with and without facial movements indicates that these movements are insufficient to explain the differences, and are likely not contaminating the results of experiments with all trials included, though they may be contributing to desirability-independent noise. The results of the experiment in which both rewarding and aversive stimuli were liquids (juice/vinegar) also corroborates the conclusion that the difference in hemodynamic response is not simply motion-related, since the motor responses (swallowing, occasional licking) were seen to be nearly the same for all liquids, due to the deep placement of the sipper tube in the mouth.

#### Decoding preferences in single trials

**Figure 6:**
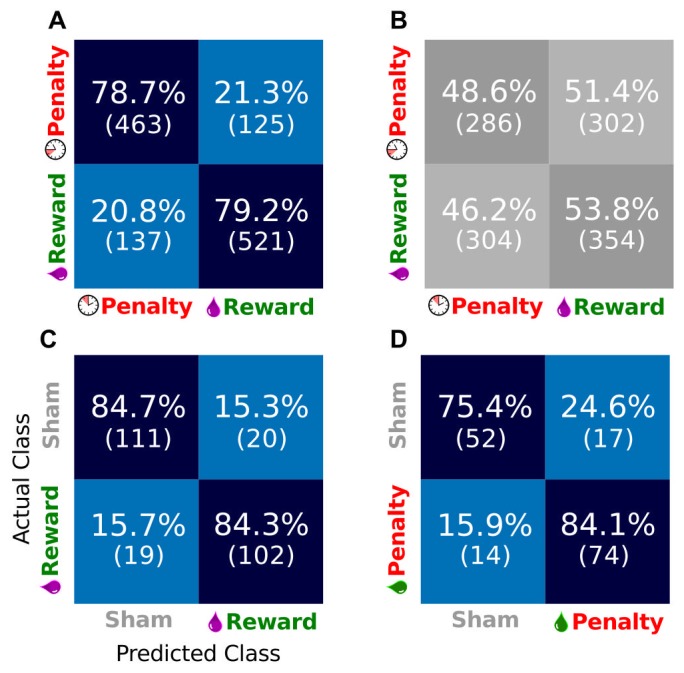
Single trial classification performance on NIRS signals. (A) Confusion matrix for test set prediction performance of SVM classifier using both Δ[HbO] and Δ[HbD] on cued trials with a single color scheme. Results are totals across 15 experimental sessions. Data used is from the cue onset to 15s-post outcome. Each box contains the percentage of test set trials in the “Actual class” that were assigned the label in the “Predicted Class” by the SVM (as labeled in panel C). Absolute numbers of trials are in parentheses. Thus, the successful classifications are on the diagonal. All other panels use the same conventions. (B) Confusion matrix for the same data as in panel A, but with the class labels shuffed. (C) Confusion matrix for classification of unexpected liquid rewards (juice) versus idle baseline (sham events). Totals are across 6 sessions. (D) Confusion matrix for unexpected penalties (vinegar) versus idle baseline (sham events). Totals are across 4 sessions.

In order to be useful as a “reward” metric for an RL algorithm, the hemodynamic signal must be resolvable at each event as signifying a relatively high or low desirability. An important component of the proposed system is therefore a classifier that is able to determine the state desirability from the NIRS signals on a single trial. A support vector machine (SVM) classifier was chosen for this purpose for its non-linearity, insensitivity to local minima, and good performance on high-dimensional problems (see Methods). All cued trials (both color significances) were classified as either reward (high desirability) or penalty (low desirability). Uncued trials were classified as either reward vs. baseline, or penalty vs. baseline. A separate classifier was trained for each experiment. All results presented represent the classifier performance on “test” data, which were not included in the training. The test data prediction confusion matrices for all experiment types are shown in Figure 6.

It is possible that the classifier was over-fitting to statistical regularities in the data set; for example if 90% of the examples in the set were rewards, then a classifier that predicted “reward” 100% of the time would show 90% performance. In order to control for this effect, a cross-validation run was performed on the dataset with all labels shuffled, thus destroying any relationship between the NIRS waveform and the label. If the above (true label) classifier was actually capturing a true relationship, then performance on the shuffled data should drop to chance. Chance level performance was observed on shuffled data (see Figure 6(B)), indicating that the unshuffled data contained a real relationship between NIRS waveform and desirability, and that the SVM was able to capture it.

#### Classifier windows

**Figure 7:**
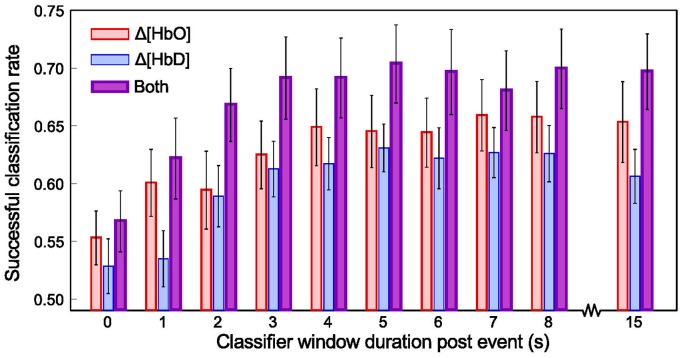
Classifier performace for different data windows and types. Mean±SEM classifier success rate (equal to the mean of the diagonal elements in the confusion matrices) across 20 experiments inluding both color conditions (n = 776 rewards; n = 683 penalties) for varying sizes of peri-event window, when using different components of the NIRS hemodynamic signal. All windows began at cue onset. Thus, the 0 window duration post event corresponds to the use of 8 seconds of data between cue onset and the outcome event. All other windows include post-outcome data.

In order to determine which components of the peri-stimulus NIRS signal were most informative about the stimulus desirability, SVM classifiers were trained and tested using only Δ [HbO], only Δ [HbD], or both, each for varying windows around the cue and outcome. All windows began at the cue onset, and ended at a time relative to the outcome delivery (see Figure 2(A)). Classifier performance was observed to increase for increasing windows past the cue delivery up to 3 seconds, after which it plateaued (Figure 7). For all windows, a trend was observed in which Δ [HbO] alone outperformed Δ [HbD] alone, and the combination of both was better than either. The improvement achieved by using both signals over using Δ [HbD] alone was significant (p<0.05) at all time windows except 0.

### RL Algorithm Applied to Virtual Task with a Noisy Reward Signal

In order to test the efficacy of the NIRS state desirability signal as a “reward” signal for a reinforcement learning agent, we programmed a model task that contained sensor readings of a simple environment, an end effector (tool) that interacted with the environment, and reward signals (see Figure 1A, and Methods section). It is important to distinguish here between the *true desirability* of the trial outcome, and the single-trial *reward signal*. The user may find an outcome truly desirable every time, but the classifier may misclassify the associated hemodynamic signal as undesirable on any given trial. The classified reward signal as presented to the agent at each trial is a realization of a probability distribution set up by the true desirability. In a realistic implementation of an RL algorithm such as 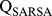 that uses a biological signal of state desirability as its reward, the decoder noise (i.e. the error in signal classification, as demonstrated above) will lead to unreliable reward information delivered to the agent. The question then arises: with the approximately 70% accuracy in determining true desirability, can a 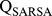 agent still converge to a reliable (state, action) value function, or will it become unstable when faced with misclassified reward or penalty events?

**Figure 8:**
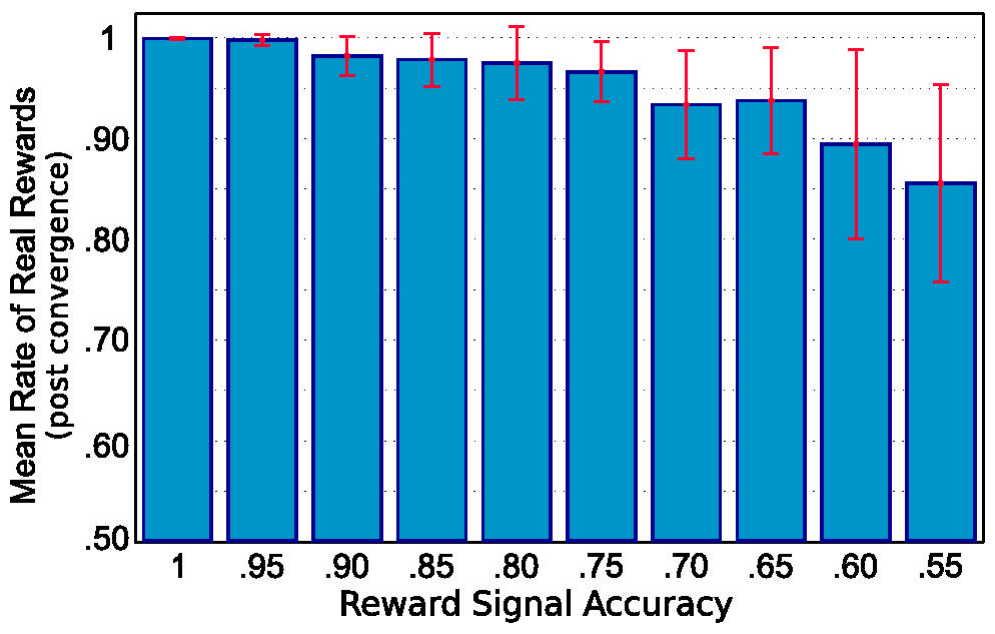
Performance of the QSARSA learner when faced with noisy reward signals. Each bar represents the results for a set of trials with feedback accuracy to the agent as indicated along the horizontal axis. Bar heights represent mean fractions of true reward outcomes (i.e. trial successes) out of 20,000 trials after convergence. Error bars are standard deviations. With increasing reward signal accuracies, the rates of reward improve and the inter-trial variance decreases.

The average performace of the 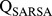 agent during the period following convergence is quite good, as seen in Figure 8. The agent comes to prefer actions that result in the truly desired outcome (pellet reaching the front of the table), in spite of the often incorrect information about its reward value. The success rate is significantly higher than the reward accuracy rate for all accuracy levels. This illustrates the ability of the agent to learn the structure of the task and find a good solution even when the reward signal is unreliable. It achieves this by aggregating a weighted average of the reward signal over time, assigning credit for new rewards based on the number of times each (state, action) pair has been visited previously. This reduces the influence of later rewards, avoiding large fluctuations on receipt of rewards or penalties for each individual outcome. The value function was seen to converge after 100–1000 training trials, with shorter convergence times for higher accuracy reward signals.

## Discussion

The results presented in this paper have established the availability of reward-related information in hemodynamic signals recorded from the frontal lobe of alert primates using NIRS. The modeling results also demonstrate the feasibility of using these signals on an asynchronous trial-by-trial basis to direct the adaptation of a BMI system that employs an RL algorithm as its controller. The advantages of such a system over those previously described are two-fold: First, NIRS signals can be recorded from the cortex non-invasively, obviating the need for surgical implants that carry inherent risks and susceptibility to interface deterioration. Second, the RL framework offers a degree of adaptability that fully supervised training algorithms do not, allowing for ongoing improvement in performance, and incorporation of novel information about the enviroment, system properties, and users' desires.

### Peri-event Signals

Deviations from baseline Δ [HbO] and Δ [HbD] were observed for both primarily desirable and secondarily desirable (i.e. predictive) stimuli. Increases relative to baseline for both signals, and therefore for Δ [HbTot], were observed around desirable outcomes, but not for unpleasant outcomes. The primary difference is an early (0.5–2s post-stimulus) influx of some additional volume of oxygen-rich blood for the desirable stimuli that is absent for unpleasant stimuli (Figure 3). A decrease in the [HbD]/[HbO] ratio (as occurs with the influx of oxygenated blood) is consistent with increased input synaptic potentials to the region [67]. We believe the influx may be also be partly attributable to the direct action of dopamine on cerebral microvessels as discussed below in the section “Implications of hemodyamic signal decoding in studies of reward”.

The negative perturbation in Δ [HbO] and Δ [HbTot] between cue and outcome may reflect a decrease in neural activity relative to baseline, which may reflect a diminished need for vigilance once the outcome is determined. This interpretation is speculative, but the measured concentrations in this period do differ significantly between the reward and penalty conditions. This difference, like the more robust difference in the post-outcome period, confirm the ability of NIRS to detect hemodynamics related to stimulus desirability. The more pronounced change in response to rewarding stimuli than to penalty stimuli corresponds to an encoding of “value” based on the definitions of Roesch et al [68]. This quantity is consistent with “desirability” when dealing with passive tasks as used in this study.

Even though there is variation in the peri-event hemodynamics, their form proved sufficiently stereotypical so that a single-trial classifier was able to use them to predict the unknown desirability of the trials stimuli (see Classification discussion below). This reinforces the validity of their interpretation as markers for reward-related neural activity, and provides for their application in BMI systems. These findings correspond to previously-observed hemodynamic responses observed in the human frontal lobe with fMRI: Tobler et al. found that the DLPFC contains partially overlapping regions with significant activation correlations with reward magnitudes, reward probabilities, and their product: reward expected value [18].

Neurons encoding various aspects of reward are apparent in DLPFC [5]–[31]. Wallis and Miller showed that 66% of neurons recorded in macaque DLPFC coded parametrically for reward magnitude during the second delay epoch of a two-epoch memory reward preference task [69]. This is compared to only 31% of neurons in OFC, a region more traditionally believed to encode reward magnitudes for decision-making. It should be emphasized that these signals are only one type out of many modalities of information that DLPFC and OFC neurons are observed to encode simultaneously. In the Wallis 2003 study, many of these same neurons also encoded visual stimulus location and identity, and selected eye movement direction. Nonetheless, their firing certainly does modulate with reward magnitude [38], and in other studies, DLPFC neurons have been shown to modulate with reward type [5].

In the present study, hemodynamic changes were shown to have repeatable time courses around the outcome stimulus presentation times. Most importantly, they were shown to differentiate between desirable and undesirable stimuli. Though these signals have been observed with other methods, this is the first demonstration of their detectability with NIRS in an awake, alert non-human primate. The hemodynamic desirability signals thus defined provide a set of physiological contextual states in which future studies of neural activity may be interpreted. The separation of neural ensemble activity in one region according to the concomitant hemodynamic state in the same or other regions may provide new insight into the means by which decision-related information modulates neural computation.

#### NIRS artifacts

A significant issue with the application of NIRS to the study of brain function is the possibility of contamination of the signals with artifacts due to motion [70]. These arise because loss of good contact with the scalp may allow either ambient light or light from the sources that has not passed through tissue to enter the detectors. A number of adaptive filtering algorithms have been proposed to correct for such that use either the NIRS data itself [70]–[71] or data from accelerometers affixed to the NIRS probes [72].

In the current study, the head fixation and cranially affixed probe guides are believed to be sufficient to minimize the effects of motion artifact, but a separate analysis was carried out as a further verification. The rationale for the analysis is that if the differences observed between desirable and undesirable stimuli were created by drinking-related motion artifacts in the NIRS signal then if the trials with facial motion detectable on video are analyzed separately from those with no apparent motion, the difference should disappear for the trials with no motion (and perhaps be more pronounced for those trials with motion). In fact, the opposite pattern was found: trials without motion still showed robust differences, while those with significant facial movements showed decreased separation, likely due to increased noise in the data. This supports the conclusion that while facial movements may degrade the data quality in these experiments, they are not the source of the separability between desirable and undesirable trials.

This conclusion is further supported by the opposite direction of the post-outcome changes in Δ [HbO] and Δ [HbD]. If the changes were systematically related to the loss of contact between the optical probes and the tissue, it would be expected that they would be in the same direction for both wavelengths of light used, and thus would affect the two computed concentration changes in the same way. Furthermore, the difference between pleasant and unpleasant liquid stimuli (Figure 3) argues against the presumed hemodynamic changes being motion artifact, since the facial movements were similar (swallowing, licking) in response to both types of stimuli.

Other artifacts in NIRS studies may arise due to the serial autocorrelations intrinsic to biological systems, such as heart rate, respiratory rhythm, or slow oscillations in blood pressure (Meyer waves [73]). In the present analysis, the preprocessing included band-pass filtering between 0.01 and 1 Hz, a range that is expected minimize the signal power due to heart rate (70–250 BPM = 1.16–4.16Hz). The event-related study design is also expected to normalize out variation due to mean respiratory rate (37±6 breaths/min [74]) or cyclical BP changes, since events occur at random phases of these cycles.

### Classification of Single Trial State Desirabilities

The SVM classifier correctly predicted the desirable or undesirable nature of the outcomes associated with NIRS waveforms with : 70% accuracy when using post-outcome data of 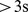. The success of the SVM classifier when using the concentration changes Δ [HbO] and Δ [HbD] in predicting the single trial significance in trials to which the classifier is naïve means that the classifier is able to capture a true relationship between hemodynamics and stimulus desirability. The SVM classifier performed, on average, equally well when using a linear kernel and when using an optimal-width radial basis function kernel (data not shown), which suggests that complex transformations of feature space do not improve accuracy. Thus, the simpler quicker method of SVM classification in linear space is preferred. Such a classifier can be trained rapidly (and thus retrained, should performance levels change over time), making its use in an online BMI application a realistic possibility. By classifying single event outcomes as desirable or undesirable, this system could serve as an online monitor of subjects' satisfaction with the performance of a neural prosthesis. This type of application is illustrated by the 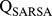 agent learning to perform the model rake task, discussed below.

Though the SVM classifier correctly classified the majority of cued trials when given access to all data throughout the trial, when it was restricted to using only pre-outcome data it made more errors (50–60% accuracy). This is above chance level, suggesting that some information about conditioned stimuli was available in the NIRS signal but is not very robust. This may be partly attributed to the task design, in which the animal had access to outcome-predicting information throughout the pre-outcome interval and it therefore required minimal recruitment of working memory during this phase. Working memory tasks are known to particularly engage lateral prefrontal activity during delay periods in which subjects must maintain working representations of task choices and possible outcomes [38]. It would be reasonable to expect such task to show better delay-period discriminability than was observed in the current experiments. As assessed in this study, however, the most robust classification requires access to outcome-related data, approaching its peak performance when using at least 3 seconds of post-outcome hemodynamic signals.

#### Limitations

One limitation to these results comes from the event-related decoding method. The event times are known to the classifier *a priori*. Thus, this method does not provide a continuous stream of information about state desirability, which would serve as an even better reinforcer for series of related actions or their constituents. This is not prohibitive for use in a BMI, however, since updates to the agent need not be applied at every time step; 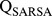 works with asynchronous updating. When the agent requires updating (due to performace dropping below a certain level, for example), events could be generated and evaluated as described. The agent would then adapt its value estimates and maintain them until another update is required.

An issue that is possibly more restrictive is the non-specific nature of the reward signals obtained in this work. These signals represent the subject's overall satisfaction with the outcomes of actions, and do not differentiate between successes or failures due to the correct execution of motor commands and those due to environmental conditions. Thus, an action that is performed correctly, but results in a penalty because of environmental factors outside the subject's control would result in negative reinforcement. This is not necessarily bad, as adaptation to environmental contingencies is one of the purposes of RL, but by adopting terminal definitions of success and failure, the method does not provide for improvement of motor behavior when it does not have extrinsically rewarding consequences. In human subjects, it is expected that proper execution of a movement would be desirable even in the absence of an immediate external reward. This would allow for specificity in adaptation based on the subject's own goals during training. However, the inverse situation may be more problematic. That is, if an external reward is achieved in spite of inaccurate motor controller output (due to pure luck), and the subject finds it satisfactory, the controller output would be reinforced. This system would be best trained when the subjects are performing tasks with explicit goals, whose fullfilment roughly parallels the accuracy of the motor output. Once trained, however, the adaptation rate could be diminished or eliminated until a new round of training is required.

#### Implications of hemodyamic signal decoding in studies of reward

Another finding of this study comes from the results of classification based on the separate Δ [HbO] and Δ [HbD] signals when compared with classification results using both chromophores together (Figure 7). Neither signal alone yielded test data prediction performance as good as the two yielded in combination. This finding is particularly interesting for its implications for the interpretation of fMRI data, which is based on the concentration changes of Δ [HbD] alone [75]. The high spin state of iron conjugated by the heme molecule (S = 2) makes HbD paramagnetic [76]. HbO, with spin state S = 0, is diamagnetic. The fMRI signal is only sensitive to paramagnetic species. The HbD signal therefore gives an incomplete picture of cerebral hemodynamics. For example, if Δ [HbD] is seen to increase, this may have been the result of decreased inflow of oxygenated arterial blood (presumably related to a regional decrease in metabolic demand due to neural activity), or the result of increased oxygen demand leaving a smaller fraction of the blood hemoglobin in the oxygenated state (presumably due to an increase in regional neural activity). Though models of cerebral blood flow help, sampling HbD alone cannot completely distinguish these states, whereas sampling HbD and HbO together can. The present classification results exploit this. Note that when the SVM classifier is given Δ [HbO] and Δ [HbD], the value of total hemoglobin Δ [HbTot] (the additive product of the two) is available implicitly in feature space. By informing the classifier of both the Δ [HbO] and Δ [HbD] signals, the decoded desirabilities may therefore have a higher correspondence with the underlying neural metabolic dynamics, and thus a higher accuracy.

There is an interesting line of evidence that dopamine acts directly on cerebral microvasculature via D1 and D5 receptors [77]–[78] [79]. This relatively recent finding may also contribute to the tighter correspondence with presumed desirability representation of the complete hemoglobin concentration signal versus the single species signals alone. It has been the basis for a call for reevaluation of the fMRI results of reward-related experiments [79]. The present results corroborate these claims, indicating that there is significant information about a cognitive variable (desirability) captured by the synergy of both components of hemoglobin dynamics, above and beyond that available in the HbD signal alone. This more complete picture of the regional hemodynamics likely corresponds more closely to the true neural activity (and thus the perceptual judgements), particulary when it involves dopamine as reward-related neural activity usually does.

Dopamine binding receptors located in cerebral microvessels (and, to a lesser degree, capillaries), could possibly be inducing an anticipatory perfusion increase to support an expected increase in neural activity by ensembles concerned with processing particularly salient information. Dopaminergic terminals are observed opposed to cortical parenchymal mirovessel (penetrating arteriole) smooth muscle cells and pericytes. Positive hemodynamic changes in frontal cortex, striatum, and thalamus are induced by dopamine releasing drugs and dopamine reuptake blockers as well as by D1/D5 receptor agonists. These positive changes are NO-independent and are mediated through activation of D1/D5 receptors, which have been observed in capillaries as well [79]. The influence of dopamine on the cortical microvascular bed creates a nonlinear effect superimposed on the tissue-oxygen-demand regulation of CBF that is not accounted for by standard impulse response models of neurovascular coupling. This direct vascular effect of dopamine may affect the interpretation of studies of reward processing based on Δ [HbD] alone.

### Model Control Task Discussion

The computational model rake task is meant to be an illustration of the type of task that a reinforcement learning BMI might be called upon to perform. The agent had to acquire knowledge of the correct sequence of actions to perform based only on updates about its environment, rather than any explicit specification of the purpose or proper execution of the task. The agent only had access to three pieces of information. The first was the pellet location on the table. The second was the direction and distance from the rake tool to the pellet. Such “difference vectors” between the end-effector (usually the hand) and a target for reaching are well known to be encoded by neural activity in the posterior parietal cortex [80]–[81]. These neural representations can even remap to use a different end-effector interaction point to compute difference vectors when using a tool [81] like the rake in the present model. The third piece of information the RL agent has access to is the reward signal, which is used to reinforce or inhibit its choices among actions. It is this reward signal component that the current simulations were designed to test. In particular, we wanted to determine whether the agent could still converge on a successful action sequence when faced with uncertain reward signals. Since the SVM classifier is only able to provide : 70–80% feedback accuracy on cued trials (Figure 6A), we wanted to test the 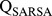 algorithm's robustness to such degraded reward signals.

In the simulations, the pellet reaching the front of the table resulted in the largest reward signal most often, and so the agent came to prefer trajectories that had this result. The expected value of the reward signal is thus seen to converge on the true desirability, and the controller exploited this property, yielding a high rate of truly rewarding outcomes: over 0.9 (see Figure 8) when using a reward signal accuracy 0.75 as per Figure 6A. The 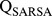 agent is able to overcome the unreliability of a realistically noisy classifier.

The 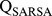 algorithm's successful performance of the model task depended on its ability to make use of delayed rewards, a significant number of which were erroneous. The learning from delayed rewards is a product of the incremental updates to values according to the TD(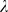) rule. The ability to deal with uncertain rewards is based on the annealing of the 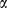 parameter with repeated exposure to (state, action) pairs (see Methods; [58]). This creates a reward-sampling effect, in which recently accumulated rewards influence the value estimation less than prior rewards. Over time, this procedure behaves with increasing momentum, responding less to individual events than to the overall trends. The result is that the algorithm converges on a solution that yields the most reward return on average. It is also notable that a simple modification of the reward landscape to include small penalties at every time step encouraged faster solutions (data not shown). This highlights the fact that useful behavioral modification of an RL agent is easily promoted by simple changes to the reinforcement signal.

As formulated here, the model task had 1183 possible 3-dimensional states, and 4 possible actions. This is a fairly large space over which the RL algorithm was able to search for solutions successfully. It seems reasonable to expect similar algorithms to deal well with the similarly large numbers of states and action possibilities that would be encountered in real applications, such as robotic limb control or computer interface operation.

Continuous state learning by such algorithms is possible too, by using function approximation to generalize value functions across regions of (state, action) space that have not been explicitly tested. This represents a merging of unsupervised learning (RL) with supervised learning (function fitting), and can be quite powerful, though often difficult to implement (see [58]).

#### Desirability signals and reinforcement learning BMIs

Reinforcement Learning attempts to determine the optimal actions that should be taken by an agent that operates in an environment with defined rewards. These algorithms are semi-supervised, requiring no explicit information about the correct output to perform effectively. Generally, RL systems include a specification of rewards in the environment, the policy followed by the agent, and a value function maintained by the agent. The policy is a function that maps states onto actions. The goal of the RL algorithm is to find the optimal policy for the agent to employ as it reads states and chooses actions in its environment.

The results presented in this paper provide for the reinforcement component for this kind of system. It should be emphasized that they are part of a larger concept, and do not provide all the requirements for a practical BMI. The method described allows for evaluative feedback from the user to the controller about actions that the controller has taken. A complete BMI will require a means for interpreting the user's intentions. That is, the system needs a way for the user to specify the timing of actions (i.e. initiate or restrain movements), as well as a practical way of defining specific intentional states. Due to the vast space of possible actions and higher-order goals, it will likely be necessary to provide some information about intended movements to the agent as states. For example, the agent would treat the state space in which the user is trying to tie their shoes differently from the state space in which the user is trying to catch a ball. Then within this set of restricted state spaces the adaptive RL algorithm may be able to refine the movements by choosing actions that maximize the user's satisfaction. To this end, a particularly useful set of state spaces would be based on decoded cortical neuronal ensemble firing patterns (similar to more traditional BMIs), and the set of actions can be based on the capabilities of a prosthetic device. This way a user could specify situation-specific goals (each state space would define its own set), and then provide feedback to the controller as it attempts to reach them. The results of the present study show that a hemodynamic signal of frontal lobe estimates of state desirability may serve as useful reinforcers for such an agent. This would form a complete system that uses CNS signals to learn and adapt a useful mapping from neural commands to prosthetic outputs.

### Conclusions

This study demonstrates a system by which hemodynamic signals of stimulus desirabilities recorded from the prefrontal cortex with NIRS may be used as reinforcers for the behavior of an adaptive BMI controller. Such a system would allow the BMI to modify its behavior over time, always pursuing mappings from inputs (neural data and artificial environmental sensor readings) to outputs (computer or prosthetic) that are as satisfactory to the user as possible. The classification and simulation results described illustrate the feasibility of the conceptual framework, and highlight the need for continued investigation into improved neural decoding for full online conscious control. They also bring into view a particular case in which the complete hemodynamic signal is capable of providing more information about a neural computation than either of its constituents alone. This has implications for future hemodynamic studies of reward and dopamine-related neural phenomena.
