# Correction: Use of Frontal Lobe Hemodynamics as Reinforcement Signals to an Adaptive Controller

**DOI:** 10.1371/annotation/d72e0974-f492-4fa4-a850-6dd0017395b5

**Published:** 2013-12-10

**Authors:** Marcello M. DiStasio, Joseph T. Francis

Part of Figure 1B was erroneously cropped out. Please see the corrected Figure 1 here: http://plosone.org/corrections/pone.0069541.g001.cn.tif

